# Saving Death: Apoptosis for Intervention in Transplantation and Autoimmunity

**DOI:** 10.1080/17402520600834704

**Published:** 2006

**Authors:** Alice Li, Okechukwu Ojogho, Alan Escher

**Affiliations:** 1 Center for Transplant Immunology Research Loma Linda University & Medical Center Loma Linda CA 92354 USA; 2 Department of Biochemistry and Microbiology School of Medicine Loma Linda University Loma Linda CA 92354 USA; 3 Transplantation Institute Loma Linda University Medical Center Loma Linda CA 92354 USA

## Abstract

Long considered immunologically “bland,” apoptotic cells are now recognized as important modulators of immune responses. The role of apoptosis in immunological homeostasis has been inferred from several findings, for example, induction of tolerance after injection of apoptotic cells and the capacity of APCs like macrophages and DCs to induce and maintain tolerance after phagocytosis of dead cells. Processing of apoptotic cells by DCs is of particular interest, because DCs are the only known APCs capable of activating naïve T lymphocytes to become effector or regulatory cells. In that regard, recent evidence suggests that phagocytosis of apoptotic cells by DCs can induce Tregs, a finding that has significant implications for the treatment of a variety of immune-mediated inflammatory disorders. Here, we review the relationship between apoptotic cells, DCs, and Tregs, and its impact on prevention of transplant rejection and treatment of autoimmune diseases.


*Clinical & Developmental Immunology*, June–December 2006; 13(2–4): 273–282

Taylor & Francis

Taylor & Francis Group

### Saving death: Apoptosis for intervention in transplantation and autoimmunity

ALICE LI[1,2], OKECHUKWU OJOGHO[1,3], & ALAN ESCHER[1,2]

[1]*Center for Transplant Immunology Research, Loma Linda University & Medical Center, Loma Linda, CA 92354, USA,* [2]*Department of Biochemistry and Microbiology, School of Medicine, Loma Linda University, Loma Linda, CA 92354, USA, and* [3]*Transplantation Institute, Loma Linda University Medical Center, Loma Linda, CA 92354, USA*

**Abstract**

Long considered immunologically "bland," apoptotic cells are now recognized as important modulators of immune responses. The role of apoptosis in immunological homeostasis has been inferred from several findings, for example, induction of tolerance after injection of apoptotic cells and the capacity of APCs like macrophages and DCs to induce and maintain tolerance after phagocytosis of dead cells. Processing of apoptotic cells by DCs is of particular interest, because DCs are the only known APCs capable of activating naïve T lymphocytes to become effector or regulatory cells. In that regard, recent evidence suggests that phagocytosis of apoptotic cells by DCs can induce Tregs, a finding that has significant implications for the treatment of a variety of immune-mediated inflammatory disorders. Here, we review the relationship between apoptotic cells, DCs, and Tregs, and its impact on prevention of transplant rejection and treatment of autoimmune diseases.

**Keywords:** *Apoptosis, tolerance, dendritic cell, regulatory T cell, transplantation, autoimmune disease*

**Abbreviations:** *AICD, activation induced cell death; ANA, antinuclear antibody; APCs, antigen presenting cells; BM, bone marrow; CFA, complete Fraud adjuvant; DCs, dendritic cells; DST, donor specific transfusion; EAE, experimental autoimmune encephalomyelitis; GAD, glutamic acid decarboxylase; GD, Graves' disease; HCs, hematopoietic cells; HT, Hashimoto's thyroiditis; MS, multiple sclerosis; NF-κB, nuclear factor-κB; NOD, non-obese diabetic; T1DM, Type 1 diabetes mellitus; TNF, tumor necrotic factor; TRAIL, TNF related apoptosis inducing ligand; Tregs, regulatory T cells*

### Introduction

Fifty to seventy billion cells die daily of apoptosis in the average human adult (Reed 1999), and for many years it was believed that these cells had little effect on the immune system. However, apoptotic cells are now thought to provide a source of antigens to establish peripheral tolerance, and can also induce strong immunogenic responses after infection by a pathogen. The remarkable capacity of apoptotic cells to induce either tolerogenic or immunogenic responses makes them attractive candidates to intervene in many diseases. Since the immune responses triggered by apoptotic cells are generally the result of their processing by APCs, it is reasonable to propose that the immunological mechanisms underlying treatments based on induction of apoptosis will closely mimic those operating in physiological immune responses. Thus, apoptotic cells could provide a means of treating diseases that is both safe and potent.

Apoptosis plays an important role in inducing and maintaining immune unresponsiveness to self-antigens via both central and peripheral tolerance. Most autoreactive T lymphocytes with high avidity for MHC-self peptide complexes are eliminated in the thymus through apoptosis (Sprent and Kishimoto 2001). However, some do exit the thymus to the

Correspondence: A. Escher, Loma Linda University, 11234 Anderson Street, Room 2576, Loma Linda, CA 92354, USA. Tel: 1 909 558 1000. Ext. 81423. Fax: 1 909 558 0486. E-mail: aescher@llu.edu

ISSN 1740-2522 print/ISSN 1740-2530 online © 2006 Taylor & Francis
DOI: 10.1080/17402520600834704

periphery, where deletion, anergy, and Tregs check their activities. It is clear that a certain level of control is mediated by APCs, which include DCs that have processed apoptotic cells generated under steady-state conditions (Chernysheva et al. 2002; Chung et al. 2006). After phagocytosis, the DCs migrate to local draining lymph nodes where they present self-antigens to T lymphocytes to induce and maintain tolerance (reviewed by Skoberne et al. 2005). Similar events have been observed when using apoptosis for induction of peripheral tolerance to treat pathogenic, immune-mediated inflammations. In this article, we review and discuss recent concepts relating to immunoregulatory apoptosis, and its role in the induction of peripheral tolerance for intervention in transplantation and autoimmune diseases.

### Apoptosis and DCs

Under steady-state conditions, internalization by peripheral DCs of self-antigens carried by apoptotic cells induces tolerance that protects cells and tissues from the damages of pathogenic autoimmunity as well as immune responses induced by viral and bacterial infections (Skoberne et al. 2005). The same mechanism may be invoked to explain antigen-specific tolerance induced by apoptotic cells that contain autoimmune and donor antigens in models of pathological autoimmunity and transplantation. A wide variety of parameters are likely to be involved in determining whether a DC becomes tolerogenic or immunogenic after uptake of apoptotic cells. For example, it is known that early stage compared to late stage apoptotic cells are more likely to induce tolerance (Sauter et al. 2000; Chernysheva et al. 2002; Ip and Lau 2004), and that molecules displayed on the surface of apoptotic cells (Skoberne et al. 2006), number of apoptotic cells (Ronchetti et al. 1999), receptor and cytokine milieu (Chen et al. 2001a,b; Albert 2004), presence or absence of danger signals (Feng et al. 2003), and interaction with other cells (Skoberne et al. 2005) can all contribute to determine different types of immune response. In addition, the state of DC maturity can play a role in the induction of tolerogenicity or immunogenicity. Immunogenic responses are generally associated with mature DCs, which display high numbers of MHC-II and co-stimulatory molecules. However, it is more difficult to establish a correlation between the maturity state of a DC and its tolerance-inducing function. Early evidence indicated that tolerance in the periphery is controlled by immature DCs (Steinman et al. 2000). However, it is becoming clear that semi-mature and mature DCs can also induce antigen-specific tolerance (Chernysheva et al. 2002; Lutz and Schuler 2002; Verhasselt et al. 2004). Moreover, tolerogenic DC do not appear to be restricted to a specific cell lineage, because both plasmacytoid and myeloid DCs have been reported to be tolerogenic (Fugier-Vivier et al. 2005; MacDonald et al. 2005; Steptoe et al. 2005). This remarkable plasticity of DCs is likely a reflection of the crucial importance of maintaining immune tolerance and controlling damages inflicted by immunogenic responses under a variety of circumstances.

Dendritic cells can induce tolerance through several mechanisms after engulfing antigen-loaded apoptotic cells. Steinman and colleagues found that capture by DCs of dying syngeneic $TAP^{-/-}$ splenocytes loaded with small amount of ovalbumin (OVA) results in OVA-specific deletional tolerance *in situ* (Bonifaz et al. 2002). A single injection of small amounts of OVA ($<150$ ng/mouse) deletes more than a million antigen specific $CD8^+$ T cells. After uptake of OVA-loaded dying cell by $CD8^+$ splenic DCs, OVA-specific $CD8^+$ T lymphocytes from transgenic mice are driven into cell cycle, deleted, and the animals become tolerant to OVA rechallenge. Moreover, when DCs are matured after exposure to anti-CD40 antibodies, the same approach causes immunity towards OVA with increased production of IL-2 and IFN-$\gamma$. Similarly, Kusuhara and co-workers have reported that OVA-pulsed, CD95 ligand-transduced killer DCs cause rapid apoptosis of OVA specific naïve $CD4^+$ T cells *in vitro* and *in vivo* (Kusuhara et al. 2002), while Min et al. (2001) have reported that FAS ligand-transfected DCs induce apoptosis of antigen-specific $CD8^+$ T cells. Other data also indicate that immature DCs can deliver apoptotic signals to antigen-specific T cells, and cause them to undergo abortive proliferation and deletion (Iyoda et al. 2002; Clayton et al. 2003; Morelli et al. 2003; Fugier-Vivier et al. 2005). Figure 1 depicts the possible interactions between DCs and Tregs and the different events involving apoptotic cells.

Remarkably, apoptotic DCs by themselves appear to play a significant role in induction of tolerance. Chen et al. (2006) have shown in a transgenic mouse model that expression of the baculovirus caspase inhibitor p35 by DCs results in their accumulation, chronic lymphocyte activation, and autoimmunity indicated by increasing antinuclear antibodies (ANA) in mice older than 9 months. In contrast, transgenic mice expressing p35 in their T or B cells do not generate ANA, indicating a definite role of apoptotic DCs in maintenance of tolerance. In another study, it was shown that apoptotic DCs are responsible for antigen-specific tolerance induced after injection of apoptotic splenocytes (Maeda et al. 2005). A potential explanation for these results is that apoptotic DCs can no longer upregulate co-stimulatory molecules to an immunogenic level, but still retain their tolerogenic APC function.

### Apoptosis and Tregs

Little is known regarding a possible link between phagocytosis of apoptotic cells and induction of Tregs.

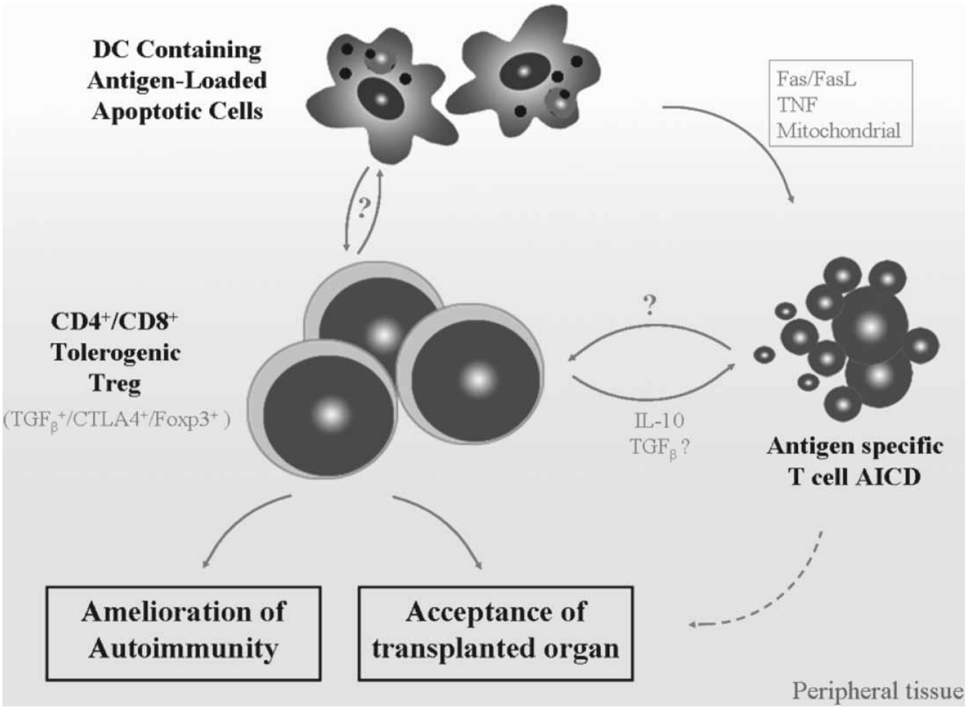

Figure 1. Dendritic cells as inducers for antigen specific tolerance. Phagocytosis of antigen-carrying apoptotic cell by peripheral DCs can induce antigen-specific tolerance. Whether the outcome is tolerance or immunity likely depends on many factors, such as the physiological conditions, type of phagocyte, nature of receptors and cytokines, site of phagocytosis, and interaction with other cells, for example, regulatory T cells. Although immature DCs are the most effective cells in this process, semi-mature and even mature DCs can be tolerogenic under certain conditions. Moreover, both plasmacytoid and myeloid DCs can be tolerogenic. Such DCs may also deliver apoptotic signals, instead of activation signals, to antigen-specific T cell clones and induce abortive proliferation as well as subsequent deletion.

Nonetheless, there has been a rapid growth in the knowledge of the bidirectional relationship between DCs and Tregs, including the effects of tolerogenic DCs that process apoptotic cells on Tregs. For example, DC-expanded, antigen-specific Tregs show greatly enhanced efficacy relative to polyclonal populations in blocking experimental autoimmunity, indicating a functional relationship between DCs and Tregs (Tarbell et al. 2006).

Considering the importance of active suppression in maintaining tolerance, it is reasonable to propose that the constant homeostatic flux of apoptotic cells generated *in vivo* could result in induction of Tregs (Ferguson et al. 2002; Barratt-Boyes and Thomson 2005; Morelli 2006).

Evidence suggesting a possible role for Tregs in apoptosis-induced tolerance was obtained from studies of Treg-associated cytokine secretions, which have been reviewed elsewhere (Aubin and Mousson 2004). For example, loading immature DCs with apoptotic splenocytes induces cardiac allograft tolerance accompanied by secretion of anti-inflammatory IL-10 (Xu et al. 2004). Further, over-expression of IL-10 in the cardiac allograft after gene transfer augments Fas- and Bax-mediated apoptosis of graft-infiltrating $CD4^+$ and $CD8^+$ T lymphocytes (Tung et al. 2003).

In addition, other investigators have shown that intravenous infusion of apoptotic splenocytes either donor, third party, or even of recipient origin, together with allogeneic BM graft can induce humoral immune tolerance in sublethal-irradiated recipient mice (Perruche et al. 2004). The induced humoral tolerance shows as a near absence of antibodies-mediated allo-cytotoxicity, together with an immune deviation shifted from type 1 to 2 response, which exhibited as anti-donor cytotoxic antibodies ($IgG_{2a}$) decreased to normal level compared to non-apoptotic infusion BM recipient controls. Such an apoptotic cell induced humoral tolerance is $TGF_\beta$ dependent, suggesting a possible role for Tregs, but has no effect on the BM graft alone control even with a higher BM cell number.

More direct evidence for induction of Tregs after apoptotic cell infusion comes from a study of self-leukocyte extracorporeal photopheresis using PUVA (UVA plus psoralen, such as 8-methoxypsoralen, a DNA-intercalating agent acting as photosensitizer). In that study, PBMC from four individually organ-transplanted infants were exposed to PUVA *in vitro* and re-injected at regular intervals. Photopheresis was shown to cause equally apoptosis of T, B, and NK cells, as well as monocytes in the PBMC. Using an *in vitro* test, DCs isolated following PUVA treatment were shown to remain immature and to phagocytose apoptotic cells. Using an *in vivo* test, Tregs were shown to be the only cell population with significant increase and suppressive function following treatment. Most importantly, the PUVA-induced increase in Treg activity lasted up to 1 year after the last exposure in 50% of the patients (Lamioni et al. 2005). Other investigators have also shown that intravenous infusion of apoptotic splenocytes into mice directly induces suppressive Tregs that are TGF-β-dependent, Foxp3$^+$, CD62L$^{high}$, and CTLA-4$^+$ (Kleinclauss et al. 2006). However, in that model system, BM graft tolerance induction is mediated by host macrophages, but not by host DCs or donor phagocytes.

Additional information on Treg induction by apoptotic cells comes from mouse models of hapten and contact hypersensitivity (reviewed by Barratt-Boyes and Thomson (2005) and Morelli (2006)). Infusion of PUVA-treated and pooled splenocytes and lymph node cells into syngeneic naïve mice causes inhibition of hapten immune response (Maeda et al. 2005). Inhibition depends on CD11c$^+$ DCs in the donor cell population and induces antigen-specific CD4$^+$CD25$^+$ Tregs in the recipient. Deleting the CD11c$^+$ population, but not T cells, in the PUVA treated donor cells, causes loss of tolerance in the recipients. Hapten tolerance can be maintained in recipient mice after adoptive transfer, but only when the transferred leukocyte pool contains CD4$^+$CD25$^+$ Tregs which release IL-10 and TGF-β. These results underscore the notion that DCs and Tregs, respectively, induce and maintain tolerance after exposure to apoptotic cells.

In animal models of autoimmune diseases, Tregs play a major role in maintaining antigen-specific tolerance to self, although evidence suggests that Tregs likely act at a later phase in the genesis of disease (Marleau and Sarvetnick 2005). As in the case of transplantation, data indicate that induced apoptosis has the potential to activate antigen-specific Tregs and ameliorate pathological autoimmunity. Indeed, work done in our laboratory shows that a pro-apoptotic DNA vaccine encoding BAX and a secreted form of the β cell antigen GAD recruits DCs and induces foxp3$^+$ Tregs in the NOD mouse model for type 1 diabetes (Li et al. 2004, 2006). Dendritic cells isolated from treated mice can suppress T cell proliferation after pulsing with GAD polypeptide (unpublished results), and Tregs cultured from pancreatic draining lymph node under GAD pulse show suppressive function and express the *foxp3* gene (Li et al. 2006). These data suggest that induction of apoptosis *in vivo* can result in antigen-specific tolerance, most likely mediated by DCs and Tregs. Additional data indicate that antigen-specific foxp3$^+$ Tregs induced by GAD-containing apoptotic cells may have developed from CD4$^+$CD25$^-$ foxp3$^-$ T cell populations (Li et al. 2006). However, the relationship between apoptotic cell-induced DCs and antigen-specific Tregs remains to be established *in vivo* in this model system. Interestingly, reports from other laboratories indicate that AICD of effector T cells is required for the induction of suppressive Tregs in autoimmune tolerance models, but the relationship between DCs, Tregs, and AICD remains obscure. CD4$^+$CD25$^+$ Tregs are particularly sensitive to AICD, which results in increased or decreased CD4$^+$CD25$^+$ Tregs activity and cell number (Chen and Brosnan 2006). Activated CD4$^+$CD25$^+$ Tregs themselves can control autoimmunity by inducing AICD (Madakamutil et al. 2003). For example, immunization with mycobacterial preparation Bacille Calmette-Guerin (BCG) prevents the onset of diabetes in NOD mice by inducing TNF-α/IFN-γ-induced apoptosis of diabetogenic T cells through both Fas and TNF pathways (Qin et al. 2004). In other examples, a MHC II-peptide octamer of HA antigen delays the onset of diabetes in TCR-CD4/RIP-HA double transgenic mice via clonal deletion of diabetogenic CD4 T cells in both thymus and peripheral lymphoid organs through AICD, while the MHC II-peptide dimmer of HA protects against diabetes by induction of IL-10 secreting Tr-1 cells in the pancreas (Preda-Pais et al. 2005), and treatment of DCs with 1α, 25-Dihydroxyvitamin D3 or its analogue plus autoantigen (GAD or insulin) selectively induces apoptosis of human autoreactive T cell clones (van Halteren et al. 2004). Altogether, these data suggest a relationship between Tregs and apoptotic cells that can lead to tolerance (Figure 2).

In another animal model of pathogenic autoimmunity, Madakamutil and co-workers have reported that expansion of CD4$^+$ and CD8$^+$ Tregs results in apoptotic deletion of Vβ8.2$^+$T cells reactive to the autoantigen myelin, and protection from EAE *in vivo* (Madakamutil et al. 2003). In addition, Ferguson et al. (2002) have shown that by using i.v. injection of apoptotic trinitrophenyl (TNP)-coupled splenocytes as tolerogen, uptake of the apoptotic tolerogen-coupled cells by CD8$^+$CD11c$^+$ DCs cross-present antigen in a MHC I-dependent manner to regulatory CD8$^+$T cells, and induces immune unresponsiveness. A subsequent study showed that i.v. injection of soluble peptide antigen-OVA emulsified 1:1 in CFA caused deletion of a large number of antigen specific DO11.10-transgenic T cells by CD8$^+$ cytotoxic T cells via predominately Fas/FasL apoptotic mechanism, resulting in antigen-specific tolerance *in vivo* (Herndon et al. 2005). Altogether, these results suggest that there may be an additional level of control at play when relatively large numbers of peripheral T cells become apoptotic, i.e. active immunoregulation can be directed toward antigen-reactive cells to control self-responses when large numbers of T cells die and release potentially dangerous autoantigens and cytokines.

This body of evidence indicates that it is possible to induce antigen-specific Tregs *in vivo* to delete autoreactive cell clones and ameliorate disease in

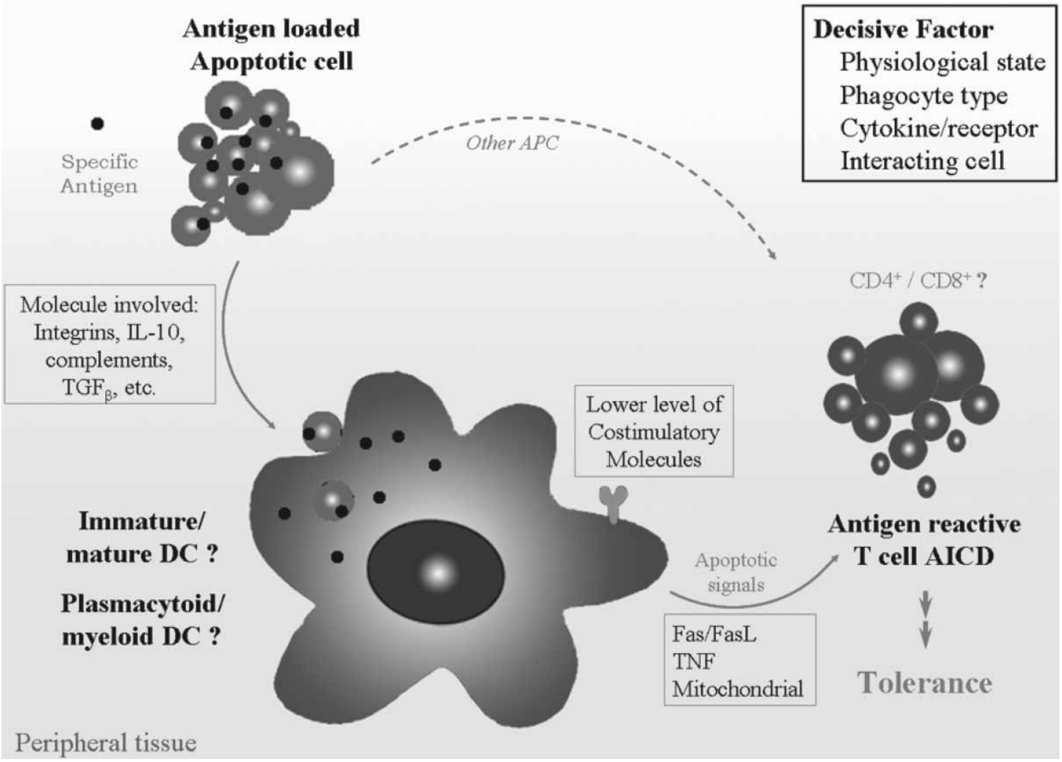

Figure 2. Regulatory T cells as maintainers of antigen-specific tolerance. While DCs play a major role in inducing antigen specific T cell apoptosis in an early phase of transplant rejection or autoimmunity, Tregs are necessary for maintaining tolerance over the long term. Activated by DCs, $CD4^+$ and $CD8^+$ Tregs monitor the periphery and keep specific antigen reactive T or B lymphocytes under control via inactivation through cell-to-cell contact, secretion of cytokines, and apoptosis to protect tissues and organs from immune rejection and autoimmunity.

animal models of autoimmune disease. The induced $CD4^+CD25^+$ Tregs may act as an initial suppression, and then induce secondary suppression by activating Tr1 or Th3 cells to extend tolerance to other population of lymphocytes (Stassen et al. 2004). However, a clear link between apoptosis-tolerized DCs, antigen-specific Tregs, and deletion of autoreactive cells remains to be firmly established.

### Apoptosis for intervention in transplantation: Application to mixed chimerism

Combined infusion of HCs and solid organ transplantation, which is performed together with non-myeloablative regimens or less intensive conditions, is one of the most promising research directions for induction of antigen-specific transplant tolerance (Ferrand et al. 2003). Full chimerism, where the hematopoietic system of the recipient is replaced by that of the donor following ablative conditioning, is associated with high morbidity and mortality that limit its application in the clinic. In contrast, mixed chimerism refers to a state where allogeneic HCs coexist with recipient cells, which can be induced in adult animals by administering T cell-depleted allogeneic and host marrow cells following high doses of total lymphoid irradiation, or recently even with non-myeloablative regimens (Sykes 2001) by using DST. The term "macrochimerism" is used to describe the persistence of high levels of donor cells in recipients, as observed after allogeneic HCs transplantation, and "microchimerism" is refer to the persistence of a small number of donor cells that are detectable using molecular techniques (Ferrand et al. 2003).

Mixed chimerism offers significant advantages over full chimerism: (1) it is less toxic; (2) it can establish tolerance across complete MHC barriers, which is a basic requirement for organ transplantation; and (3) because of the coexistence of host- and donor-derived hematopoietic APC in the thymus, intrathymic deletion of host-reactive cells in addition to donor-reactive cells occurs in mixed chimeras to a greater extent than in full chimeras. The minimal, nontoxic host conditioning regimens for mixed chimerism induction offers a hopeful future for clinical transplantation. In that regard, evidence indicates that delivery of apoptotic cells is a promising means to help establishing mixed chimerism.

Infusion of apoptotic HCs is thought to have the potential to induce robust chimerism, reduce the requirements for immunosuppressors, and allow the simultaneous performance of the HCs infusion with grafting, rather than before grafting (Kleinclauss et al. 2003b), which improves the clinical applicability remarkably. Indeed, and as previously mentioned, Saas and colleagues have shown that an infusion of apoptotic mononuclear cells from different sources can enhance tolerance to an allogeneic graft (Bittencourt et al. 2001). The investigators used intravenous injection of apoptotic cells in combination with allogeneic bone marrow cells to induce macrochimerism and increase the success rate of bone marrow transplantation (Kleinclauss et al. 2003a; Perruche et al. 2003).

With the emerging role of Tregs in strategies where apoptotic or non-apoptotic HCs infusion is combined with organ transplantation, a link between Tregs and establishment of chimerism has been postulated. When only low level of donor chimerism persists, for example, when high doses of HCs are given with non-myeloablative regimens in solid organ transplantation, maintenance of unresponsiveness can be achieved through additional mechanisms, such as suppression mediated by Tregs, to clear or inhibit remaining donor-reactive T or B lymphocytes (Kurtz et al. 2004; Saas et al. 2004; Sykes et al. 2005). However, recent reports have shown that, although $CD4^{+}CD25^{+}$ Tregs may not be necessarily involved in the induction of chimerism, they do play a role in the maintenance of stable mixed chimerism established through BM transplant with costimulatory blockade (CTLA4Ig and anti-CD154) plus rapamycin in a non-myeloablative skin-grafted mouse model (Zheng et al. 2003; Domenig et al. 2005).

Observations made in the clinic can also help us to understand further the role of apoptosis in induction of transplant tolerance. For example, a group of patients receiving kidney–pancreas transplant with no rejection, under an immunosuppressive regimen consisting of anti-thymoglobulin antibodies for 7 days and cyclosporine/mofetil mycophenolate as maintenance therapy, have shown higher intragraft apoptosis (annexin V) and lower anti-apoptotic molecules (lymphocytes CD95L, iNOs, and Bcl-2) together with higher CD8, CD4DR expression in graft and higher CD3CD25 expression in PBL compared to healthy controls (Fiorina et al. 2004). However, the controls from the same organ transplanted with rejections were not compared in this report. In another study, a comparison between acute non-rejected and rejected human orthotopic liver transplantations showed that non-rejecting subjects had higher donor cell chimerism detected by PCR on day 0, and higher apoptotic recipient PBMC with increase expression of IFNγ, IL-10, and CD40L on day 1 when compared to acutely-rejected patients (Jonsson et al. 2004). In the case of the unexpected restoration of cardiac function via stem cell transplantation after myocardial infarctions, it has been proposed that apoptosis of transplanted stem cells modulate local immune reactivities by down-regulating innate and adaptive immunity (Thum et al. 2005). This may have occurred through deactivation of macrophages and DCs, as well as stimulation of Tregs, which could have led to the observed reduced scar formation, repressed myocardial apoptosis, and improved cardiac outcome. These data indicate that induced or infused apoptotic cells have significant potential for future clinical applications.

A drawback associated with using infused apoptotic cells is the limited supply of donor BM or HC cells that could be used as apoptotic cells in the clinic. Therefore, it will be important to identify alternative sources of apoptotic cells. For example, iliac crest, progenitor from PBMC, cultured $CD34^{+}$ stem cells, and $NK1.1^{+}$ $TCR\alpha\beta^{+}$ BM cells all have potential as replacements for induction of chimerism using tolerogenic apoptosis (Delis et al. 2006).

### Apoptotic cells for intervention in autoimmune diseases

Dysregulated apoptosis, whether interrupted or accelerated, is thought to be a central defect in many human and murine autoimmune diseases. Defective apoptosis can contribute to different etiological aspects of autoimmune disease, such as interruption of central or peripheral T cell death that can lead to AICD failure and increased numbers of autoreactive cells, and disruption of organ-specific cell apoptosis that leads to autoimmune reactivities (Kuhtreiber et al. 2003; Todaro et al. 2004). In the NOD mouse model for type 1 diabetes, it was shown that down regulation of LMP2/TAP1 molecules may be responsible for impaired assembly of self peptide antigen-loaded MHC I molecules, lead to faulty activation of pro-inflammatory transcription factors such as NF-κB, and result in impaired self-education of DCs and T lymphocytes as well as increased sensitivities of lymphocytes to TNF-α induced apoptosis (Kuhtreiber et al. 2003). In addition, a NOD-specific, TCR-induced, AICD-resistance profile has been reported in $CD4^{+}$ and $CD8^{+}$ T cells. Upon activation by anti-CD3 antibodies, the NOD-derived T cells are more resistant to AICD compared to C57BL/6, BALB/c, and NOR T cells, accompanied with lower expression of Fas and FasL, caspase 3 and 8 levels, IL-2 and IL-4, and higher TGF-β and Bclx-L levels (Arreaza et al. 2003; Decallonne et al. 2003). Furthermore, Fas functional defect is also observed in human T1DM patients (Dianzani et al. 2003). The apoptotic defects in different cell types and the consequent defective T cell AICD could be responsible for defective peripheral tolerance and development of T1DM.

Different laboratories have shown that induction of apoptosis can prevent or treat T1DM in animal models. Induction of TNF-α expression after administration of

CFA combined with re-education of newly emerging T cells with self-antigen can interrupt autoimmune diabetes in NOD mice through the loss of cells with an increased sensitivity to TNF-α—induced apoptosis, leading to long-term tolerance (Ryu et al. 2001). Similarly, a transgenic mouse model expressing islet-specific TNF-α abrogates T1DM through increasing apoptosis of autoreactive $CD8^{+}$ lymphocytes at a time when the disease is already ongoing (Christen and Von Herrath 2002). In addition, nicotinamide, a poly (ADP-ribose) polymerase inhibitor, is thought to prevent spontaneous and recurrent diabetes in NOD mice by inducing apoptosis of islet-infiltrating leukocytes (Suarez-Pinzon et al. 2003).

Among the different pro-apoptotic strategies for prevention or treatment of type 1 diabetes, DNA vaccination could represent the simplest and most practical means to intervene. As mentioned previously, we have reported that a DNA vaccine encoding both a secreted form of the β-cell antigen GAD and pro-apoptotic BAX in order to generate apoptotic cells containing GAD antigen can prevent autoimmune diabetes when delivered intramuscularly (Li et al. 2004), and ameliorate new-onset diabetes when injected intradermally in NOD mice (Li et al. 2006). We have proposed that the vaccine triggers formation of GAD-containing apoptotic bodies *in vivo* that induce tolerogenic DCs and Tregs. BAX in this model can be taken as an adjuvant to promote the prophylactic and therapeutic effects of the vaccine, similarly to the apoptotic effect of vaccine adjuvants that has been reported as an important factor for induction of the differentiation and maturation of DCs and activation of T cells (Yang et al. 2004). However, BAX can also be used as an adjuvant to induce immunogenic response (Kinsey et al. 2004). Indeed, our own data indicate that, in contrast with a secreted GAD vaccine, a plasmid encoding higher amounts of intracellular GAD protein does not prevent diabetes and is pro-inflammatory (Li et al. 2004). A possible hypothesis that could explain these different results is that the lower amounts of GAD contained in apoptotic bodies do not induce over expression of molecular chaperones and other stress proteins that are thought to act as danger signals (Feng et al. 2003; Masse et al. 2004). Low amounts of GAD antigen could maintain "subimmunogenic" conditions that are known to favor conversion of naïve T cells into Tregs (Kretschmer et al. 2005), and result in tolerance for the antigen contained in apoptotic cells. In addition to using DNA vaccination to induce apoptotic cells as a source of tolerogenic antigen, a different pro-apoptotic DNA vaccination strategy has used plasmid DNA encoding antigen (i.e. beta-galactosidase) and FasL to induces antigen-specific peripheral T cell tolerance through the induction of FasL-mediated antigen specific AICD (Georgantas et al. 2000).

In the central nervous system, T cell AICD-resistance is also considered important in the etiology of EAE and MS, and induced apoptosis of CNS-infiltrating lymphocytes improves the disease (Pender 1999; Pender and Rist 2001; Chen and Brosnan 2006). Intrathecal delivery of IFN-γ protects mice from chronic progressive EAE associated with increased apoptosis of CNS-infiltrating lymphocytes (Furlan et al. 2001). In addition, 1, 25-dihydroxy-vitamin D3 reverses EAE by stimulating apoptosis in CNS inflammatory cells such as macrophages and $CD4^{+}$T cells (Spach et al. 2004).

In murine or human autoimmune arthritis, defective apoptosis of synoviocytes, T, and B lymphocytes has been reported to be associated with disease onset, characterized by chronic inflammatory cell infiltration and synovial hyperplasia (Mountz et al. 2001; Zhang et al. 2001; Lopez-Hoyos et al. 2003; Peng 2006). Several interventions using induction of Fas or non-Fas apoptotic pathway in different cell types have been shown to be beneficial in RA models, which have been reviewed previously (Peng 2006). Recently, direct evidence has shown that repeated injection of FasL encoded by an adenovirus increases the number of apoptotic cells in arthritic synovium and dramatically reduced the size and weight of the synovium. This approach has been named "gene scalpel" in reference to synovectomy but without surgical damaging (Zhang et al. 2005). In addition, intra-articular injection of TRAIL, which induces apoptosis in proliferating cells, encoding adenovirus or TRAIL protein induces extensive synovial lining apoptosis as well as decreasing inflammation in rabbit arthritis models (Yao et al. 2006).

There is also evidence that autoimmune thyroiditis, such as HT and GD, are apoptosis-related diseases. Comparative research of human HT and GD shows that in HT thyroid, the regulation of Fas/FasL/Bcl-2 favors thyrocyte apoptosis via homophylic Fas–FasL interactions and a gradual reduction in thyrocyte numbers leading to hypothyroidism. In contrast, the regulation of Fas/FasL/Bcl-2 in GD favors apoptosis of infiltrating lymphocytes and thus favors thyrocyte survival and hyperthyroidism (Salmaso et al. 2002). Induced apoptosis targeting different cell types in HT or GD could have therapeutic effects on both of the two forms of thyroid autoimmunity.

### Conclusion

We are starting to recognize the fundamental importance of apoptotic cells for the maintenance of immune homeostasis, and to use apoptosis in a rational manner to ameliorate diseases. However, it is clear that a variety of molecular and cellular parameters can greatly impact the immune response induced by apoptosis. Considering that dying cells can induce either tolerogenic or immunogenic responses, it will be vital to identify and control the conditions that determine these two different outcomes if apoptosis is to be used as an intervention in a clinical setting.

Direct delivery of apoptotic cells will require induction of apoptosis *in vitro* using UV, chemical, or even genetic means before infusion, with the benefit of knowing the number and quality of the cells to be delivered. Induction of apoptosis directly *in vivo* appears more practical, could permit tissue-specific induction of apoptosis, and be achieved using similar means. However, the quantity and quality of the induced apoptotic cells will be more difficult to evaluate. According to different needs, protocols could be designed to use one or both strategies to achieve optimal results. Because of its potential safety and potency, therapeutic or even prophylactic induction of apoptosis could offer a wide range of applications for maintenance of tolerance in the fields of graft transplantation, pathological autoimmunity, and other immune-mediated inflammatory disorders.

### References

Albert ML. 2004. Death-defying immunity: Do apoptotic cells influence antigen processing and presentation? Nat Rev Immunol 4:223–231.

Arreaza G, Salojin K, Yang W, Zhang J, Gill B, Mi QS, Gao JX, Meagher C, Cameron M, Delovitch TL. 2003. Deficient activation and resistance to activation-induced apoptosis of CD8+ T cells is associated with defective peripheral tolerance in nonobese diabetic mice. Clin Immunol 107:103–115.

Aubin F, Mousson C. 2004. Ultraviolet light-induced regulatory (suppressor) T cells: An approach for promoting induction of operational allograft tolerance? Transplantation 77:S29–S31.

Barratt-Boyes SM, Thomson AW. 2005. Dendritic cells: Tools and targets for transplant tolerance. Am J Transplant 5:2807–2813.

Bittencourt MC, Perruche S, Contassot E, Fresnay S, Baron MH, Angonin R, Aubin F, Herve P, Tiberghien P, Saas P. 2001. Intravenous injection of apoptotic leukocytes enhances bone marrow engraftment across major histocompatibility barriers. Blood 98:224–230.

Bonifaz L, Bonnyay D, Mahnke K, Rivera M, Nussenzweig MC, Steinman RM. 2002. Efficient targeting of protein antigen to the dendritic cell receptor DEC-205 in the steady state leads to antigen presentation on major histocompatibility complex class I products and peripheral CD8+ T cell tolerance. J Exp Med 196:1627–1638.

Chen L, Brosnan CF. 2006. Exacerbation of experimental autoimmune encephalomyelitis in P2X7R−/− mice: Evidence for loss of apoptotic activity in lymphocytes. J Immunol 176:3115–3126.

Chen W, Frank ME, Jin W, Wahl SM. 2001a. TGF-beta released by apoptotic T cells contributes to an immunosuppressive milieu. Immunity 14(6):715–725.

Chen Z, Moyana T, Saxena A, Warrington R, Jia Z, Xiang J. 2001b. Efficient antitumor immunity derived from maturation of dendritic cells that had phagocytosed apoptotic/necrotic tumor cells. Int J Cancer 93:539–548.

Chen M, Wang YH, Wang Y, Huang L, Sandoval H, Liu YJ, Wang J. 2006. Dendritic cell apoptosis in the maintenance of immune tolerance. Science 311(5764):1160–1164.

Chernysheva AD, Kirou KA, Crow MK. 2002. T cell proliferation induced by autologous non-T cells is a response to apoptotic cells processed by dendritic cells. J Immunol 169:1241–1250.

Christen U, Von Herrath MG. 2002. Apoptosis of autoreactive CD8 lymphocytes as a potential mechanism for the abrogation of type 1 diabetes by islet-specific TNF-alpha expression at a time when the autoimmune process is already ongoing. Ann NY Acad Sci 958:166–169.

Chung EY, Kim SJ, Ma XJ. 2006. Regulation of cytokine production during phagocytosis of apoptotic cells. Cell Res 16:154–161.

Clayton AR, Prue RL, Harper L, Drayson MT, Savage CO. 2003. Dendritic cell uptake of human apoptotic and necrotic neutrophils inhibits CD40, CD80, and CD86 expression and reduces allogeneic T cell responses: Relevance to systemic vasculitis. Arthritis Rheum 48:2362–2374.

Decallonne B, van Etten E, Giulietti A, Casteels K, Overbergh L, Bouillon R, Mathieu C. 2003. Defect in activation-induced cell death in non-obese diabetic (NOD) T lymphocytes. J Autoimmun 20:219–226.

Delis S, Burke GW 3rd, Ciancio G. 2006. Bone marrow-induced tolerance in the era of pancreas and islets transplantation. Pancreas 32:1–8.

Dianzani U, Chiocchetti A, Ramenghi U. 2003. Role of inherited defects decreasing Fas function in autoimmunity. Life Sci 72:2803–2824.

Domenig C, Sanchez-Fueyo A, Kurtz J, Alexopoulos SP, Mariat C, Sykes M, Strom TB, Zheng XX. 2005. Roles of deletion and regulation in creating mixed chimerism and allograft tolerance using a nonlymphoablative irradiation-free protocol. J Immunol 175:51–60.

Feng H, Zeng Y, Graner MW, Likhacheva A, Katsanis E. 2003. Exogenous stress proteins enhance the immunogenicity of apoptotic tumor cells and stimulate antitumor immunity. Blood 101:245–252.

Ferguson TA, Herndon J, Elzey B, Griffith TS, Schoenberger S, Green DR. 2002. Uptake of apoptotic antigen-coupled cells by lymphoid dendritic cells and cross-priming of CD8(+) T cells produce active immune unresponsiveness. J Immunol 168:5589–5595.

Ferrand C, Perruche S, Robinet E, Martens A, Tiberghien P, Saas P. 2003. How should chimerism be decoded? Transplantation 75:50S–54S.

Fiorina P, Torriani G, Gremizzi C, Davalli AM, Orsenigo E, Bruno Ventre M, Dell'Antonio G, Carlo VD, Rossini S, Secchi A. 2004. Selective intra-graft apoptosis and down-regulation of lymphocyte bcl-2, iNOs and CD95L expression in kidney-pancreas transplanted patients after anti-thymoglobulin induction. Transpl Int 17:603–608.

Fugier-Vivier IJ, Rezzoug F, Huang Y, Graul-Layman AJ, Schanie CL, Xu H, Chilton PM, Ildstad ST. 2005. Plasmacytoid precursor dendritic cells facilitate allogeneic hematopoietic stem cell engraftment. J Exp Med 201:373–383.

Furlan R, Brambilla E, Ruffini F, Poliani PL, Bergami A, Marconi PC, Franciotta DM, Penna G, Comi G, Adorini L, Martino G. 2001. Intrathecal delivery of IFN-gamma protects C57BL/6 mice from chronic-progressive experimental autoimmune encephalomyelitis by increasing apoptosis of central nervous system-infiltrating lymphocytes. J Immunol 167:1821–1829.

Georgantas RW 3rd, Leong KW, August JT. 2000. Antigen-specific induction of peripheral T cell tolerance *in vivo* by codelivery of DNA vectors encoding antigen and Fas ligand. Hum Gene Ther 11:851–858.

Herndon JM, Stuart PM, Ferguson TA. 2005. Peripheral deletion of antigen-specific T cells leads to long-term tolerance mediated by CD8+ cytotoxic cells. J Immunol 174:4098–4104.

Ip WK, Lau YL. 2004. Distinct maturation of, but not migration between, human monocyte-derived dendritic cells upon ingestion of apoptotic cells of early or late phases. J Immunol 173:189–196.

Iyoda T, Shimoyama S, Liu K, Omatsu Y, Akiyama Y, Maeda Y, Takahara K, Steinman RM, Inaba K. 2002. The CD8+

dendritic cell subset selectively endocytoses dying cells in culture and *in vivo*. J Exp Med 195:1289–1302.

Jonsson JR, Gu W, Vanags DM, Bishop GA, McCaughan GW, Fawcett J, Lynch SV, Balderson GA, Powell EE, Clouston AD. 2004. Increased mononuclear cell activation and apoptosis early after human liver transplantation is associated with a reduced frequency of acute rejection. Liver Transpl 10: 397–403.

Kinsey BM, Marcelli M, Song L, Bhogal BS, Ittmann M, Orson FM. 2004. Enhancement of both cellular and humoral responses to genetic immunization by co-administration of an antigen-expressing plasmid and a plasmid encoding the pro-apoptotic protein Bax. J Gene Med 6:445–454.

Kleinclauss F, Bittard H, Perruche S, de Carvalho-Bittencourt M, Chalopin JM, Herve P, Tiberghien P, Saas P. 2003a. Tolerance in transplantation: Potential contribution of haematopoietic transplantation and cell therapy. Prog Urol 13:1406–1414.

Kleinclauss F, Perruche S, Cahn JY, Tiberghien P, Saas P. 2003b. Administration of donor apoptotic cells: An alternative cell-based therapy to induce tolerance? Transplantation 75:43S–45S.

Kleinclauss F, Perruche S, Masson E, de Carvalho Bittencourt M, Biichle S, Remy-Martin JP, Ferrand C, Martin M, Bittard H, JM, Seilles E, Tiberghien P, Saas P. 2006. Intravenous apoptotic spleen cell infusion induces a TGF-beta-dependent regulatory T-cell expansion. Cell Death Differ 13:41–52.

Kretschmer K, Apostolou I, Hawiger D, Khazaie K, Nussenzweig MC, von Boehmer H. 2005. Inducing and expanding regulatory T cell populations by foreign antigen. Nat Immunol 6:1219–1227.

Kuhtreiber WM, Hayashi T, Dale EA, Faustman DL. 2003. Central role of defective apoptosis in autoimmunity. J Mol Endocrinol 31:373–399.

Kurtz J, Wekerle T, Sykes M. 2004. Tolerance in mixed chimerism—a role for regulatory cells? Trends Immunol 25:518–523.

Kusuhara M, Matsue K, Edelbaum D, Loftus J, Takashima A, Matsue H. 2002. Killing of naive T cells by CD95L-transfected dendritic cells (DC): *In vivo* study using killer DC-DC hybrids and CD4(+) T cells from DO11.10 mice. Eur J Immunol 32:1035–1043.

Lamioni A, Parisi F, Isacchi G, Giorda E, Di Cesare S, Landolfo A, Cenci F, Bottazzo GF, Carsetti R. 2005. The immunological effects of extracorporeal photopheresis unraveled: Induction of tolerogenic dendritic cells *in vitro* and regulatory T cells *in vivo*. Transplantation 79:846–850.

Li A, Ojogho O, Franco E, Baron P, Iwaki Y, Escher A. 2006. Pro-apoptotic DNA vaccination ameliorates new onset of autoimmune diabetes in NOD mice and induces foxp3+ regulatory T cells *in vitro*. Vaccine 24: 5036–5046.

Li AF, Hough J, Henderson D, Escher A. 2004. Co-delivery of pro-apoptotic BAX with a DNA vaccine recruits dendritic cells and promotes efficacy of autoimmune diabetes prevention in mice. Vaccine 22:1751–1763.

Lopez-Hoyos M, Marquina R, Tamayo E, Gonzalez-Rojas J, Izui S, Merino R, Merino J. 2003. Defects in the regulation of B cell apoptosis are required for the production of citrullinated peptide autoantibodies in mice. Arthritis Rheum 48:2353–2361.

Lutz MB, Schuler G. 2002. Immature, semi-mature and fully mature dendritic cells: Which signals induce tolerance or immunity? Trends Immunol 23:445–449.

MacDonald KP, Rowe V, Clouston AD, Welply JK, Kuns RD, Ferrara JL, Thomas R, Hill GR. 2005. Cytokine expanded myeloid precursors function as regulatory antigen-presenting cells and promote tolerance through IL-10-producing regulatory T cells. J Immunol 174:1841–1850.

Madakamutil LT, Maricic I, Sercarz E, Kumar V. 2003. Regulatory T cells control autoimmunity *in vivo* by inducing apoptotic depletion of activated pathogenic lymphocytes. J Immunol 170:2985–2992.

Maeda A, Schwarz A, Kernebeck K, Gross N, Aragane Y, Peritt D, Schwarz T. 2005. Intravenous infusion of syngeneic apoptotic cells by photopheresis induces antigen-specific regulatory T cells. J Immunol 174:5968–5976.

Marleau AM, Sarvetnick N. 2005. T cell homeostasis in tolerance and immunity. J Leukoc Biol 78:575–584.

Masse D, Ebstein F, Bougras G, Harb J, Meflah K, Gregoire M. 2004. Increased expression of inducible HSP70 in apoptotic cells is correlated with their efficacy for antitumor vaccine therapy. Int J Cancer 111:575–583.

Min W, Huang X, Gorczynski R, Cattral M. 2001. Fas ligand-transfected dendritic cells induce apoptosis of antigen-specific T cells. Transplant Proc 33:234.

Morelli AE. 2006. The immune regulatory effect of apoptotic cells and exosomes on dendritic cells: Its impact on transplantation. Am J Transplant 6:254–261.

Morelli AE, Larregina AT, Shufesky WJ, Zahorchak AF, Logar AJ, Papworth GD, Wang Z, Watkins SC, Falo LD Jr, Thomson AW. 2003. Internalization of circulating apoptotic cells by splenic marginal zone dendritic cells: Dependence on complement receptors and effect on cytokine production. Blood 101: 611–620.

Mountz JD, Hsu HC, Matsuki Y, Zhang HG. 2001. Apoptosis and rheumatoid arthritis: Past, present, and future directions. Curr Rheumatol Rep 3:70–78.

Munoz LE, Gaipl US, Franz S, Sheriff A, Voll RE, Kalden JR, Herrmann M. 2005. SLE—a disease of clearance deficiency? Rheumatology (Oxford) 44:1101–1107.

Pender MP. 1999. Activation-induced apoptosis of autoreactive and alloreactive T lymphocytes in the target organ as a major mechanism of tolerance. Immunol Cell Biol 77:216–223.

Pender MP, Rist MJ. 2001. Apoptosis of inflammatory cells in immune control of the nervous system: Role of glia. Glia 36:137–144.

Peng SL. 2006. Fas (CD95)-related apoptosis and rheumatoid arthritis. Rheumatology (Oxford) 45:26–30.

Perruche S, Kleinclauss F, Angonin R, Cahn JY, Deconinck E, Reininger L, Boucraut J, Tiberghien P, Saas P. 2003. A single intravenous infusion of apoptotic cells, an alternative cell-based therapy approach facilitating hematopoietic cell engraftment, did not induce autoimmunity. J Hematother Stem Cell Res 12:451–459.

Perruche S, Kleinclauss F, Bittencourt Mde C, Paris D, Tiberghien P, Saas P. 2004. Intravenous infusion of apoptotic cells simultaneously with allogeneic hematopoietic grafts alters anti-donor humoral immune responses. Am J Transplant 4:1361–1365.

Preda-Pais A, Stan AC, Casares S, Bona C, Brumeanu TD. 2005. Efficacy of clonal deletion vs. anergy of self-reactive CD4 T-cells for the prevention and reversal of autoimmune diabetes. J Autoimmun 25:21–32.

Qin HY, Chaturvedi P, Singh B. 2004. *In vivo* apoptosis of diabetogenic T cells in NOD mice by IFN-gamma/TNF-alpha. Int Immunol 16:1723–1732.

Ronchetti A, Rovere P, Iezzi G, Galati G, Heltai S, Protti MP, Garancini MP, Manfredi AA, Rugarli C, Bellone M. 1999. Immunogenicity of apoptotic cells *in vivo*: Role of antigen load, antigen-presenting cells, and cytokines. J Immunol 163: 130–136.

Reed JC. 1999. Dysregulation of apoptosis in cancer. J Clin Oncol 17:2941–2953.

Ryu S, Kodama S, Ryu K, Schoenfeld DA, Faustman DL. 2001. Reversal of established auto-immune diabetes by restoration of endogenous beta cell function. J Clin Invest 108:63–72.

Saas P, Kleinclauss F, Tiberghien P. 2004. Immune regulation and transplantation: An exciting challenge. Transplantation 77:S38–S40.

Salmaso C, Bagnasco M, Pesce G, Montagna P, Brizzolara R, Altrinetti V, Richiusa P, Galluzzo A, Giordano C. 2002. Regulation of apoptosis in endocrine autoimmunity: Insights from Hashimoto's thyroiditis and Graves' disease. Ann NY Acad Sci 966:496–501.

Sauter B, Albert ML, Francisco L, Larsson M, Somersan S, Bhardwaj N. 2000. Consequences of cell death: Exposure to necrotic tumor cells, but not primary tissue cells or apoptotic cells, induces the maturation of immunostimulatory dendritic cells. J Exp Med 191(3):423–434.

Skoberne M, Beignon AS, Larsson M, Bhardwaj N. 2005. Apoptotic cells at the crossroads of tolerance and immunity. Curr Top Microbiol Immunol 289:259–292.

Skoberne M, Somersan S, Almodovar W, Truong T, Petrova K, Henson PM, Bhardwaj N. 2006. The apoptotic cell receptor CR3, but not {alpha}v{beta}5, is a regulator of human dendritic cell immunostimulatory function. Blood. Epub ahead of print.

Spach KM, Pedersen LB, Nashold FE, Kayo T, Yandell BS, Prolla TA, Hayes CE. 2004. Gene expression analysis suggests that 1,25-dihydroxyvitamin D3 reverses experimental autoimmune encephalomyelitis by stimulating inflammatory cell apoptosis. Physiol Genomics 18:141–151.

Sprent J, Kishimoto H. 2001. The thymus and central tolerance. Philos Trans R Soc Lond B Biol Sci 356:609–616.

Stassen M, Schmitt E, Jonuleit H. 2004. Human $CD4^{+}CD25^{+}$ regulatory T cells and infectious tolerance. Transplantation 77:S23–S25.

Steinman RM, Turley S, Mellman I, Inaba K. 2000. The induction of tolerance by dendritic cells that have captured apoptotic cells. J Exp Med 191:411–416.

Steptoe RJ, Ritchie JM, Jones LK, Harrison LC. 2005. Autoimmune diabetes is suppressed by transfer of proinsulin-encoding Gr-1 + myeloid progenitor cells that differentiate *in vivo* into resting dendritic cells. Diabetes 54:434–442.

Suarez-Pinzon WL, Mabley JG, Power R, Szabo C, Rabinovitch A. 2003. Poly (ADP-ribose) polymerase inhibition prevents spontaneous and recurrent autoimmune diabetes in NOD mice by inducing apoptosis of islet-infiltrating leukocytes. Diabetes 52:1683–1688.

Sykes M. 2001. Mixed chimerism and transplant tolerance. Immunity 14:417–424.

Sykes M, Shimizu I, Kawahara T. 2005. Mixed hematopoietic chimerism for the simultaneous induction of T and B cell tolerance. Transplantation 79:S28–S29.

Tarbell KV, Yamazaki S, Steinman RM. 2006. The interactions of dendritic cells with antigen-specific, regulatory T cells that suppress autoimmunity. Semin Immunol 18:93–102.

Thum T, Bauersachs J, Poole-Wilson PA, Volk HD, Anker SD. 2005. The dying stem cell hypothesis: Immune modulation as a novel mechanism for progenitor cell therapy in cardiac muscle. J Am Coll Cardiol 46:1799–1802.

Todaro M, Zeuner A, Stassi G. 2004. Role of apoptosis in autoimmunity. J Clin Immunol 24:1–11.

Tung TC, Oshima K, Cui G, Laks H, Sen L. 2003. Dual upregulation of Fas and Bax promotes alloreactive T cell apoptosis in IL-10 gene targeting of cardiac allografts. Am J Physiol Heart Circ Physiol 285:H964–H973.

van Halteren AG, Tysma OM, van Etten E, Mathieu C, Roep BO. 2004. 1alpha, 25-dihydroxyvitamin D3 or analogue treated dendritic cells modulate human autoreactive T cells via the selective induction of apoptosis. J Autoimmun 23:233–239.

Verhasselt V, Vosters O, Beuneu C, Nicaise C, Stordeur P, Goldman M. 2004. Induction of FOXP3-expressing regulatory CD4pos T cells by human mature autologous dendritic cells. Eur J Immunol 34:762–772.

Xu DL, Liu Y, Tan JM, Li B, Zhong CP, Zhang XH, Wu CQ, Tang XD. 2004. Marked prolongation of murine cardiac allograft survival using recipient immature dendritic cells loaded with donor-derived apoptotic cells. Scand J Immunol 59:536–544.

Yang YW, Wu CA, Morrow WJ. 2004. Cell death induced by vaccine adjuvants containing surfactants. Vaccine 22: 1524–1536.

Yao Q, Seol DW, Mi Z, Robbins PD. 2006. Intra-articular injection of recombinant TRAIL induces synovial apoptosis and reduces inflammation in a rabbit knee model of arthritis. Arthritis Res Ther 8:R16.

Zhang H, Gao G, Clayburne G, Schumacher HR. 2005. Elimination of rheumatoid synovium *in situ* using a Fas ligand "gene scalpel". Arthritis Res Ther 7:R1235–R1243.

Zhang J, Bardos T, Mikecz K, Finnegan A, Glant TT. 2001. Impaired Fas signaling pathway is involved in defective T cell apoptosis in autoimmune murine arthritis. J Immunol 166: 4981–4986.

Zheng XX, Sanchez-Fueyo A, Domenig C, Strom TB. 2003. The balance of deletion and regulation in allograft tolerance. Immunol Rev 196:75–84.

